# Reporting the standard error of the mean: a critical analysis of three journals in manual medicine

**DOI:** 10.1186/s12998-025-00587-y

**Published:** 2025-06-04

**Authors:** Alen Manovic, Ebba Immelsjö, Iben Axen, Per J. Palmgren

**Affiliations:** 1 Hälsosam Rörlighet Naprapati, Friledningsgatan 3B, 721 37 Västerås, Sweden; 2 Muskelcentrum, Vasagatan 34, 722 15 Västerås, Sweden; 3 Unit of Intervention and Implementation Research for Worker Health, Institute of Environmental Medicine (IMM), Karolinska Institutet, Nobels Väg 13, 171 77 Solna, Stockholm, Sweden; 4 Department of Learning, Informatics, Management and Ethics (LIME), Karolinska Institutet, Widerströmska Huset, Tomtebodavägen 18A, 171 65 Solna, Stockholm, Sweden; 5 Nordic Institute of Manual Medicine, Krääftriket 23A, 114 19 Stockholm, Sweden

**Keywords:** Statistics, Statistics as a topic, Standard deviation, Standard error of the mean, Descriptive statistics, And Variability of the study sample

## Abstract

**Background:**

In the realm of biomedical research articles, authors typically utilize descriptive statistics to outline the characteristics of their study samples. The standard deviation (SD) serves to illustrate variability among the individuals in a sample, whereas the standard error of the mean (SEM) conveys the level of uncertainty associated with the sample mean’s representation of the population mean. It is not unusual for authors of scientific articles to incorrectly utilize the SEM rather than the SD when explaining data variability. This is problematic because the SEM is consistently smaller than the SD, which could cause readers to underestimate variation in the data. In medical journals, inappropriate use has been found in 14–64% of articles. Moreover, in the field of musculoskeletal health and manual medicine, there is a noticeable absence of literature on the appropriate presentation of statistics.

**Aim:**

The aim of this study was to map the frequency of inappropriate reporting of SEM in articles published over a three-year period in three prominent journals in manual medicine.

**Methods:**

In this critical analysis, all articles in three journals – *BMC Chiropractic and Manual Therapies* (CMT), *Journal of Manipulative and Physiological Therapeutics* (JMPT) and *Musculoskeletal Science and Practice: An International Journal of Musculoskeletal Physiotherapy* (MSP) – published between 2017 and 2019 were analysed based on descriptive statistics that inappropriately or vaguely reported SEMs.

**Results:**

In total, 790 articles were analysed from the three journals, 487 of which were found to report the SEM. Among these articles, we identified a frequency of 1.4% of inadequate SEM use. The investigation also showed that in 2.5% of the cases, authors did not clarify whether the ± sign presented in text, tables or figures expressed SDs or SEMs.

**Conclusion:**

There was a low frequency (1.4%) of inaccurately reported SEMs in scientific journals focusing on manual medicine, which was notably lower than studies conducted in other fields. Additionally, it was noted that in 2.5% of the articles, the ± sign was not adequately defined, which could lead to confusion among readers and hinder the interpretation of the results.

## Background

In original quantitative scientific articles, statistics are essential to reporting findings and results. However, since the 1960s, there has been a relatively high frequency of inappropriate reported statistics in various scientific journals [1–4].

Descriptive statistics summarize a sample using measures of central tendency and dispersion, as well as frequency tables and diagrams [5, 6]. Measures of central tendency, including the mean, median, and mode, represent the central point or typical value of a data set. Each measure provides unique insights and is appropriate for different types of data and situations [7, 8].

Measures of dispersion, or variability, include the standard deviation (SD), variance, interquartile range (IQR), and range [9, 10]. These measures are often presented alongside measures of central tendency to provide a comprehensive description of the data.

Inferential statistics, also known as analytical statistics, involve the use of mathematical models to make inferences about a population based on a sample [5]. A key measure in inferential statistics is the standard error, specifically the standard error of the mean (SEM), which quantifies the discrepancy between a sample mean and the population mean [11–13]. The term "standard error" generally refers to the variability of a statistic, while "standard error of the mean" pertains specifically to the mean of a data set. This study focuses on the SEM.

The SD and SEM communicate distinct types of information [14–17]:

- The SD is a descriptive statistic, a measure of spread, or the variability among individual data points within a sample.
- The SEM is an inferential statistic, a measure of precision, and indicates the uncertainty in the estimated mean of the sample, providing insight into the precision of the estimate [18].

Despite these distinctions, authors of scientific papers sometimes use standard errors such as the SEM to describe variation [19, 20].

Studies in diverse scientific fields have examined the practice of reporting standard errors to explain variability in the data [20–22]. Incorrect use of statistics has been found at an alarming rate [7, 14, 16, 19, 23–25], and impacted studies are considered to contribute to inappropriate reporting [21, 26]. In the realm of evidence-based medicine (EBM), correct statistical reporting is crucial for reliable evidence, better patient outcomes, and advancing medicine [22]. Proper statistical reporting is also crucial for authors who aspire to publish in esteemed scientific journals, as it preserves the integrity and reliability of their research, aids in the peer-review process and ensures that the research has a meaningful and ethical impact on the field [27]. Consequently, there is a need for authors to have a good grasp of the SD and SEM and for journals to provide clear guidelines regarding their use.

The use of the ± sign in scientific publications to denote SD and SEM is problematic [16]. Altman and Bland [16], highlight that the ± sign is frequently employed without clear specification, leading to reader confusion. This ambiguity can mislead readers regarding whether it denotes SD (data dispersion) or SEM (precision of the sample mean). The authors advocate for explicitly specifying the statistics associated with the ± sign to enhance clarity. Numerous journals advise against using the ± sign unless its meaning is unequivocally clear [16].

In medical disciplines such as gynaecology, anaesthesia, and cardiology, the incorrect reporting of SEM is prevalent. Nagele [21] reviewed 860 articles in four anaesthesia journals, finding that one in four studies misused the SEM. Articles often employed both SD and SEM, with SD in the text and SEM in figures. The authors recommend clearer guidelines and standardized methods for reporting variability to mitigate confusion. Ko et al. [20] found that 14% of 456 articles in gynaecological journals inaccurately reported the SEM. Studies utilizing the SEM were cited more frequently than those using the SD. Twenty-two articles reported the mean with the ± sign without specifying SD or SEM. Ko et al. concluded that accurate statistical presentation is essential to prevent misinterpretation [20]. Wullschleger and co-authors [22] analysed 441 articles from cardiovascular journals, discovering that 64% misused the SEM. They emphasize reporting confidence intervals instead of the SEM to better convey result uncertainty and suggest clearer instructions for authors [22].

Despite the extensive body of literature in musculoskeletal health and manual medicine, there is a notable absence of studies addressing the issue of inappropriate statistical reporting. To address this gap, we sought to illuminate the inappropriate reporting of SEM in manual medicine literature, thereby contributing to a more rigorous interpretation of evidence. Consequently, the aim of this study was to map the frequency of inappropriate reporting of SEM in articles published over a three-year period in three prominent journals in manual medicine.

The research inquiries posed were as follows:

- How frequently was the SEM inadequately described in selected journals focused on manual medicine?
- How did the misuse of the SEM vary among the three journals?
- To what extent were there uncertainties regarding the interpretation of the statistical concept accompanying the ± sign in data presentations in the selected journals?

## Methods

The present work is a literature study utilizing a quantitative approach, where articles published in three leading journals in manual medicine between the years 2017 and 2019 were retrospectively evaluated and critically analysed. The project originates from a student degree project conducted at the Scandinavian College of Naprapathic Manual Medicine, Stockholm, Sweden.

### Sample and data collection

Three influential journals in manual medicine were selected: *BMC Chiropractic and Manual Therapies* (CMT), *Journal of Manipulative and Physiological Therapeutics* (JMPT) and *Musculoskeletal Science and Practice: An International Journal of Musculoskeletal Physiotherapy* (MSP). The journals were selected based on recommendations from professionals, such as chiropractors and naprapaths, rather than their impact factor. The articles examined were published in the three journals during the period 2017 to 2019.

Following communication with the Karolinska Institutet University Library, a librarian gathered all articles published in the three journals over the three-year time frame.

### Data screening, selection, and analysis

#### Article screening and selection

In the initial screening stage of data analysis, a comprehensive spreadsheet was created and shared among the authors. This spreadsheet contained essential information such as the author, publication year, and journal of each article. Additionally, it included a column indicating whether the articles were selected for further analysis or excluded, along with detailed reasons for exclusion. The exclusion criteria were clearly defined and comprised case reports, brief research reports, concise communications, original studies lacking quantitative results, and review articles. Conference abstracts were also omitted from the assessment to ensure the focus remained on full-length, peer-reviewed articles.

The primary main analysis of the selected original articles involved a meticulous examination of all tables, figures, and text in the results section, specifically related to statistical reporting. Each included article was carefully screened by manually searching for keywords such as “SD” (standard deviation), “SEM” (standard error of the mean), and “ ±,” as well as terms related to descriptive statistics, including “deviation,” “error,” and “descriptive,” using the Adobe search tool function. The presence or absence of statistical concepts such as standard deviation (SD) and standard error (SEM) was thoroughly documented to ensure a comprehensive understanding of the statistical methods employed in each study.

The articles were systematically coded in a spreadsheet using binary labels to denote “Correct use of SD/SEM,” “Inappropriate use of SEM,” and “Missing SD/SEM.” In the category “Missing SD/SEM,” articles that reported inferential statistics, such as confidence intervals, without providing descriptive statistics were also included. The reason is that making inferences without descriptive statistics is considered bad practice, thus leading to uncertainty in interpretation. The inappropriate use of SEM was characterized by its application to describe the variability of the data or measurements without reference to statistical inference. Studies employing alternative measures such as median, range, and interquartile range (IQR) were categorized under “Other measures.” Articles that ambiguously used ± signs were classified as “ ± not defined.” Instances where the correct application of SD/SEM was indeterminate were labelled as “Difficult to interpret.”

Each selected article was reviewed by two independent assessors (AM and EI). In situations where the assessors had differing opinions on coding or classification, IA and PJP were consulted to help establish a consensus. Following the completion of the analysis, PJP conducted a random sampling of 20 articles per journal to validate the findings. Of the cases analysed, only one (1.7%) demonstrated slight inconsistency in the review process.

#### Statistical analysis

Spreadsheets were imported into the Statistical Package for Social Sciences (SPSS), version 25.00. As the data from the three scientific journals during the periods 2017, 2018 and 2019 consisted of categorical variables, frequencies and proportions (expressed as a percentage) were calculated. Proportional differences between the journals were tested using the Chi-square test or Fisher’s exact test (when any cell had fewer than 5 values), followed by Cramer’s V test to measure effect size. For post hoc analysis, we compared column proportions using z-tests on 3 × 2 contingency tables by extracting adjusted standardized residuals—which function as z-scores—to assess the significance of individual cells. A Bonferroni correction was applied to control for multiple comparisons; thus, *p*-values were adjusted by dividing the original alpha level (0.05) by the number of comparisons, ensuring the overall type I error rate was controlled. Probability values less than five percent (*p* < 0.05) were considered statistically significant.

## Results

**Fig. 1:**
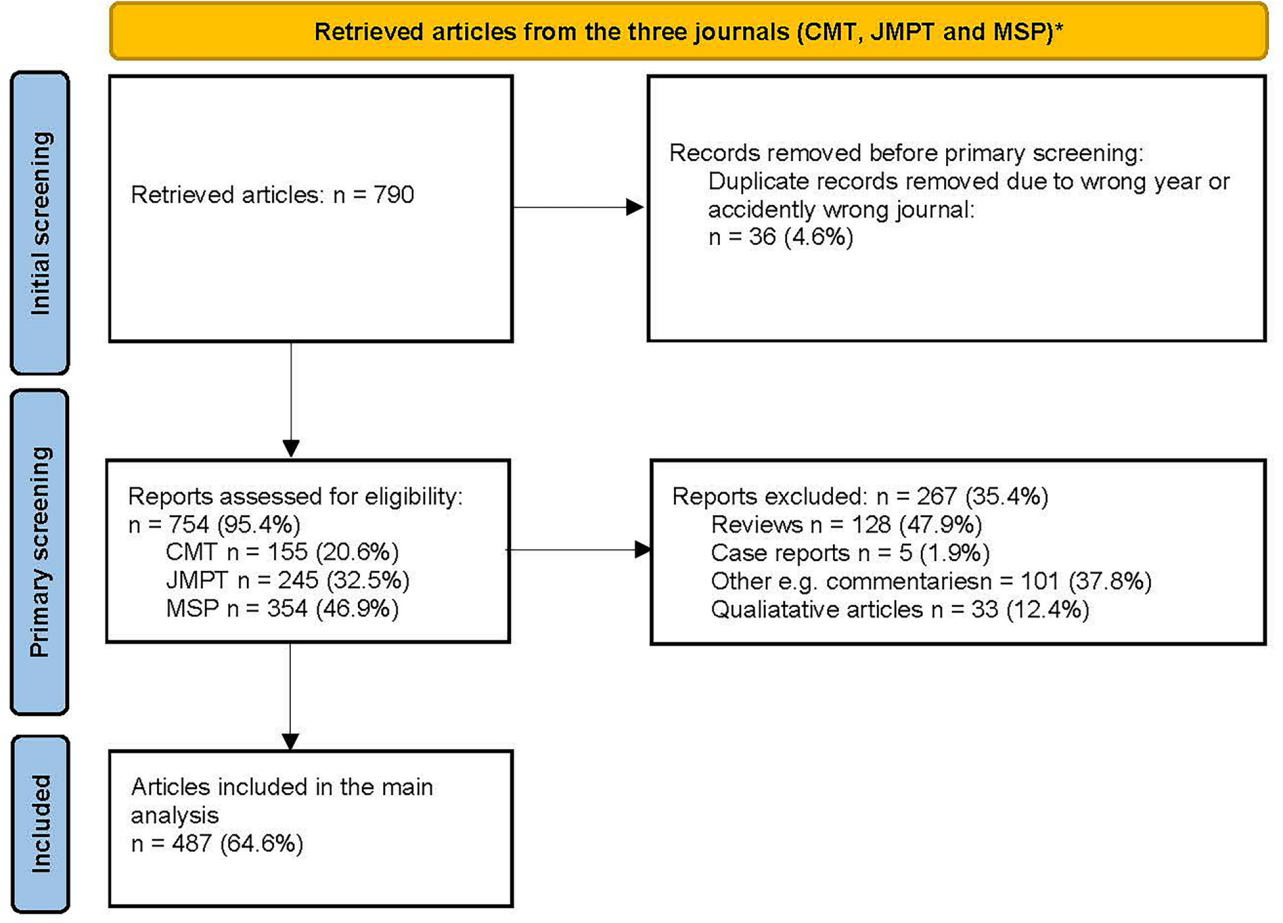
Flowchart illustrating the screening process for article inclusion and exclusion

The search retrieved 790 articles, 36 of which were excluded, as they were published in a different year (2020) or journal outside of the criteria set for the investigation. The remaining 754 articles (155 from CMT, 245 from JMPT and 354 from MSP) under scrutiny were screened, and 267 of them were excluded, as they did not meet the criteria for further analysis (see Fig. 1).

### Aggregated data for the three journals

Of the 487 included articles, the SD/SEM was correctly used in 399 (81.9%) of them, and inappropriate use of the SEM was identified in seven (1.4%). The sign ± was not defined in 12 (2.5%) articles (Table 1). There was a statistically significant association between the journal and correct use of the SD/SEM, χ^2^ = 12.9, *p* = 0.002, Cramer’s V = 0.161. Post hoc analysis indicated that the "Correct" counts for CMT (z =  − 3.5) were statistically significant. This analysis exhibited a statistically significant difference in the proportion between CMT and both JMPT and MSP, with CMT displaying a lower frequency of correct counts than expected. There were no articles in CMT that inappropriately applied the SD/SEM. In a comparison of proportional differences between correct and inappropriate uses of the SD/SEM, no significant differences were detected between the JMPT and MSP, *p* = 0.256 (Fisher’s). The frequency of not reporting the SD/SEM was notably lower in CMT. A statistically significant relationship was identified between the journal and the missing SD/SEM, χ^2^ = 15.9, *p* < 0.001, Cramer’s V = 0.181. Post hoc analysis revealed that the "Missing" counts for CMT (z = 4.0) were statistically significant. This analysis demonstrated a statistically significant difference in the proportions of not reporting the SD/SEM between CMT and both JMPT and MSP, with CMT showing a higher frequency of missing counts than expected.

**Table 1:** Aggregated data from three manual medicine journals

Journal	Correct use of SD^1^/SEM^2^	Inappropriate use of SD^1^/SEM^2^	Missing SD^1^/SEM^2^	± sign not defined^3^	Other measures^4^	Difficult to interpret
	Frequency (%)					
CMT^*^ n = 80	55 (68.8)	0	22 (27.5)	0	1 (1.3)	1 (1.3)
JMPT^*^ n = 189	157 (83.1)	5 (2.6)	20 (10.6)	9 (4.8)	3 (1.6)	1 (0.5)
MSP^*^ n = 218	187 (85.8)	2 (0.9)	24 (11.0)	3 (1.4)	0	3 (1.4)
Total n = 487	399 (81.9)	7 (1.4)	66 (13.6)	12 (2.5)	4 (0.8)	5 (1.0)

^*^CMT = *BMC Chiropractic and Manual Therapies*; JMPT = *Journal of Manipulative and Physiological Therapeutics*; and MSP = *Musculoskeletal Science and Practice: An International Journal of Musculoskeletal Physiotherapy*

^1^Standard deviation

^2^Standard error of the mean

^3^The statistical concept represented by the ± sign is not clearly explained and remains undefined

^4^Such as the median, range and interquartile range

### Data for CMT

Of the 80 screened articles, the SD/SEM was correctly used in 55 (68.8%). There were no articles with inappropriate SEM use or articles where the ± sign was identified. In one (1.3%) article, it could not be determined whether the correct or incorrect use of the SD/SEM was applied (Table 2).

**Table 2:** Tabulated data from BMC chiropractic and manual therapies (CMT)

	Correct use of SD^1^/SEM^2^	Inappropriate use of SD^1^/SEM^2^	Missing SD^1^/SEM^2^	± sign not defined^3^	Other measures^4^	Difficult to interpret
	Frequency (%)					
2017	13 (68.4)	0	5 (26.3)	0	0	1 (5.3)
2018	13 (54.2)	0	11 (45.8)	0	0	0
2019	29 (78.4)	0	6 (16.2)	0	1 (2.7)	0
Total	55 (68.8)	0	22 (27.5)	0	1 (1.3)	1 (1.3)

^1^Standard deviation

^2^Standard error of the mean

^3^The statistical concept represented by the ± sign is not clearly explained and remains undefined

^4^Such as the median, range and interquartile range

### Data for the JMPT

Among the 189 screened articles, the SD/SEM was correctly employed in 157 (83.1%). Inadequate SEM use was observed in five (2.6%) articles. The sign ± was not adequately defined in nine (4.8%) articles. In one (0.5%) article, it could not be discerned whether the correct or inappropriate use of the SD/SEM was utilized (see Table 3).

**Table 3:** Tabulated data from the journal of manipulative and physiological therapeutics

	Correct use of SD^1^/SEM^2^	Inappropriate use of SD^1^/SEM^2^	Missing SD^1^/SEM^2^	± sign not defined^3^	Other measures^4^	Difficult to interpret
	Frequency (%)					
2017	53 (80.3)	2 (3.0)	9 (13.6)	2 (3.0)	1 (1.5)	0
2018	61 (84.7)	2 (2.8)	5 (6.9)	5 (6.9)	1 (1.4)	1 (1.4)
2019	43 (84.3)	1 (2.0)	6 (11.8)	2 (3.9)	1 (2.0)	0
Total	157 (83.1)	5 (2.6)	20 (10.6)	9 (4.8)	3 (1.6)	1 (0.5)

^1^Standard deviation

^2^Standard error of the mean

^3^The statistical concept represented by the ± sign is not clearly explained and remains undefined

^4^Such as the median, range and interquartile range

### Data for MSP

In the analysis of 218 articles, 187 (85.8%) used the SD/SEM correctly, while unsuitable SEM use was present in two (0.9%). The ± sign was not properly defined in three (1.4%) articles. Furthermore, in three (1.4%) articles, it was unclear whether the correct or incorrect use of the SD/SEM was employed (Table 4).

**Table 4:** Tabulated data from musculoskeletal science and practice: an international journal of musculoskeletal physiotherapy

	Correct use of SD^1^/SEM^2^	Inappropriate use of SD^1^/SEM^2^	Missing SD^1^/SEM^2^	± sign not defined^3^	Other measures^4^	Difficult to interpret
	Frequency (%)					
2017	65 (87.8)	1 (1.4)	4 (5.4)	2 (2.7)	0	2 (2.7)
2018	57 (87.7)	1 (1.5)	7 (10.8)	0	0	1 (1,5)
2019	65 (82.3)	0	13 (16.5)	1 (1.3)	0	0
Total	187 (85.8)	2 (0.9)	24 (11.0)	3 (1.4)	0	3 (1.4)

^1^Standard deviation

^2^Standard error of the mean

^3^The statistical concept represented by the ± sign is not clearly explained and remains undefined

^4^Such as the median, range and interquartile range

## Discussion

Our study aimed to map the frequency of inappropriate reporting of SEM in three leading scientific journals within the field of manual medicine during the period from 2017 to 2019. Of the 487 articles from the three journals, inappropriate reporting of the SEM occurred in only 1.4% of them. This finding indicates that inappropriate reporting was relatively uncommon in these journals during the investigated period. Most cases of improper use of the SEM were identified in the JMPT (2.6%). In MSP, approximately one percent of the articles inadequately reported the SEM, whereas in CMT, no such instances were identified. Among the three journals examined, CMT contained the least number of articles to analyse, which potentially explains why no inappropriate reporting was found. The results of this study also indicated that in 2.5% of cases, authors failed to specify whether the ± sign appearing in the text, tables or figures denoted the SD or SEM. It is worth noting that in this study, five articles were classified as challenging to interpret, constituting one percent of the material analysed.

### Comparison with other studies

In contrast to previous studies, our results showed a lower frequency of articles where the use of the SEM was inadequate [20–22]. Nagele [21] reported that this figure was 11.5% to 27.7% in four anaesthesia journals; Ko et al. [20] identified this as 13.6% in four gynaecology journals; and Wullschleger et al. [22] reported the figure as 64% in three cardiology journals. The discrepancies in our results might be attributed to the detailed statistical guidelines provided in the authors’ instructions of the journals examined. However, upon reviewing the instructions provided by the journals currently under examination, none of the three journals explicitly delineated the difference between reporting sufficient measures of dispersion and measures of precision in their author guidelines. When juxtaposing our findings with earlier studies, it is important to note that these studies examined were based on data from 2001 [21], 2011 [20], to 2012 [22]. The differences observed in our findings compared to those of earlier research may be attributed to a heightened statistical awareness among researchers, resulting in a more accurate application of SEM during the period under study. This increased awareness might stem from a growing emphasis on statistical accuracy and rigor in the research community.

Moreover, it is conceivable that articles from the past 15–20 years have been subject to more meticulous examination during the peer-review process prior to being published. Peer reviewers and editors may have become more vigilant in ensuring the accuracy and reliability of statistical reporting, including SEM.

Furthermore, advancements in the research field may have led to a decrease in the misreporting of SEM across other medical disciplines. Improved training and education in statistics for researchers, along with the availability of better software and analytical tools, might have contributed to more accurate reporting. Despite the earlier studies with data from 2001, 2011, to 2012, and our present study, which is based on 2017–2019 data, our extensive efforts, up to the submission of this article, have not encountered any subsequent studies addressing the issue of inappropriate reporting of SEM in any medical or healthcare professional field.

The disparity in the results might also be ascribed to the fact that previous studies included a much larger number of articles. Nagele’s [21] study had the largest number of articles analysed, with 860 published across four journals over a year. However, Ko et al. [20] analysed 456 articles from four journals over a year. Lastly, Wullschleger et al. [22] examined 441 articles from three journals over a year. In our study, we analysed 487 articles over a span of three years. We assert that the findings of our study were not contingent on the quantity of articles analysed but, rather, on our methodology and an evolved understanding of statistical analysis, particularly in accurately utilizing the SEM compared to previous research.

Previous research [20, 24] has suggested that researchers may have reported the SEM instead of the SD to show “better” results with smaller spread. However, it is also possible that this subpar reporting may be due to lack of knowledge among authors regarding how to properly approach these statistical concepts.

Yet another approach distinguishing our study from previous research is that Wullschleger et al. [22] investigated the misuse of the SEM in two ways. The first approach was similar to our method of searching for statistical misreporting in descriptive statistics. The second approach was to review improper application of the SEM in inferential statistics, that is, in scenarios where one would anticipate 95% confidence intervals alongside *p*-values obtained from hypothesis testing. The latter approach was not employed in the current study.

In both Ko et al.’s [20] and Naegel´s [21] studies, articles reporting solely inferential statistics without descriptive statistics were excluded. In our analysis, we included these types of articles; however, they were noted as lacking value and denoted as “Missing SD/SEM.” This, in turn, may have distorted our findings, as more articles were included in our study, and the error percentage was consequently smaller. However, when retrospectively analysing the data, we found that articles presenting inferential statistics, such as confidence intervals, without descriptive statistics were very scarce.

In the present study, similar to those of Ko et al. [20] and Altman and Bland [16], we also identified that the ± sign often occurred without defining what it referred to. This specific issue has been a source of debate within the scientific community for a considerable period, as it may lead to confusion among readers and complicate the interpretation of research findings [10].

Journal impact factor (JIF) is a measure that provides a ratio of citations to a journal in a given year to the citable items in the prior two years [28] and can be regarded as a measure of scientific quality. The JIFs for the three journals examined in this study varied between 1.2 and 1.9. Journals examined in previous studies had impact factors between 1.8 and 4.7 [20, 21] and 5.9 and 11.6 [22]. Thus, the journals in our investigation, which had relatively low impact factors, had a comparatively low frequency of inappropriate reporting, while previous research, where journals with higher impact factors were examined, showed a higher frequency of misreporting of the SEM.

The outcomes of this study may offer novel and valuable insights into the proper reporting of the SD and SEM in research articles in the manual medicine field, as seen in the three journals from 2017 to 2019. The research revealed that these statistical concepts were appropriately utilized in these leading journals, contributing to the advancement of knowledge in manual medicine. Our work suggests that the use of the SEM in these publications was highly accurate, with minimal instances of misreporting. It is plausible that our findings reflect an enhanced awareness and adherence to statistical reporting standards among journals and researchers in the field.

### Strengths and limitations

Our work has several strengths, including filling a knowledge gap, as the use of SEM has not been previously investigated in manual medicine, unlike in other medical fields. By exploring these concepts, research article consumers may gain a better understanding of the appropriate use of SEM and its application in contemporary research. Furthermore, this work was strengthened by the clear demarcation of our analysed data, as we focused on a specific time period and successfully gathered all available data. We are confident that this approach has resulted in coherent and reproducible results.

Despite these strengths, there are several limitations to our study. Firstly, it only examined three journals. These journals were selected based on recommendations from academics and professionals within the field of manual medicine, rather than at random, which may not represent other journals in manual medicine. However, based on the SCImago Journal Rank (SJR) indicator [29], a commonly used tool in scientific writing, the three selected journals are among the top five scientific journals in the field of chiropractic and manual medicine. Secondly, when selecting the journals, we did not take their Journal Impact Factor (JIF) into account. Although it is conceivable that journals with higher impact factors place greater emphasis on the quality of statistical reporting, our results did not corroborate this assertion. Penultimately, the classification of articles depicting "inferential statistics without providing descriptive statistics" under the category "Missing SD/SEM" could be questioned. This classification was suboptimal, as it mixed different types of statistical reporting practices. For future investigations, we recommend presenting these categories separately to ensure clearer and more accurate interpretation of the data. Finally, our work is based on an undergraduate degree project carried out in 2021. If a replication study were to be conducted today using more up-to-date data, the results could potentially vary.

### Implications for future research

Based on our investigation, we believe that future studies should explore several additional aspects. First, other journals in the field of manual medicine should be examined, as well as more recent time ranges than those used in this study. To address these gaps, one of the authors (PJP) has recently launched a project examining manual medicine from 2020 to 2024, analyzing data from the top five journals by JIF and comparing these findings with those in the field of speech pathology. This comparison is motivated by the need to understand how statistical reporting practices differ across disciplines, potentially revealing unique challenges and opportunities for improving the rigor of evidence in manual medicine. Secondly, future empirical work should extend beyond simple frequency counts by employing complex statistical models, such as mixed linear models (LMM) and generalized linear mixed models (GLMMs). These models enable a more nuanced analysis of the data and provide deeper insights into the relationships between variables.

Furthermore, prior research in the field has examined the prevalence of articles that reported only SEM instead of confidence intervals [22], This topic warrants further investigation in future studies within the domain of manual medicine, as exploring the implications of using only SEM could yield valuable insights. Finally, it may also be of value to conduct a more in-depth investigation, through a critical qualitative document analysis, of the prevailing recommendations of journals within the field of manual and musculoskeletal medicine with regards to statistical reporting in scientific articles. We believe that our work can shed light on the problem of misreporting of the SEM and possibly help in further developing journal recommendations for presumptive contributors, thereby enhancing the future quality of published research.

## Conclusion

Only a small fraction (1.4%) of the articles analysed revealed inappropriate use of the SEM. This percentage is considerably lower than that reported in earlier studies across different medical fields. While the general rate of inappropriate SEM usage remains low, there were distinctions among the three journals studied. Moreover, in 2.5% of the cases, the ± sign was not included, which suggests a lack of clarity in these scientific publications.

## Data Availability

No datasets were generated or analysed during the current study.

## Abbreviations

**CMT**: BMC chiropractic and manual therapies
**EBM**: Evidence-based medicine
**IQR**: Interquartile range
**JIF**: Journal impact factor
**JMPT**: Journal of manipulative and physiological therapeutics
**MSP**: Musculoskeletal science and practice: an international journal of musculoskeletal physiotherapy
**SD**: Standard deviation
**SEM**: Standard error of the mean
**SPSS**: Statistical package for social sciences

## Declarations

### Use of generative AI for language enhancement

Since none of the authors were native English speakers, the generative AI tool, Copilot, was employed to ensure the accuracy and clarity of the language used in this manuscript. Copilot facilitated comprehensive language checks and control, thereby enhancing the overall readability and quality of the text.
